# Experiences of Everyday Discrimination by Sexual Orientation in a Large National Cohort of Female Nurses

**DOI:** 10.1080/00918369.2026.2635574

**Published:** 2026-02-26

**Authors:** Colleen A. Reynolds, Tabor Hoatson, Landon D. Hughes, Ariel L. Beccia, Alexis R. Miranda, S. Bryn Austin, Heather L. Corliss, Brittany M. Charlton

**Affiliations:** a Department of Epidemiology, Harvard T.H. Chan School of Public Health, Boston, MA, USA; b Department of Population Medicine, Harvard Pilgrim Health Care Institute, Boston, MA, USA; c Department of Population Medicine, Harvard Medical School, Boston, MA, USA; d Division of Adolescent/Young Adult Medicine, Boston Children’s Hospital, Boston, MA, USA; e Department of Social and Behavioral Sciences, Harvard T.H. Chan School of Public Health, Boston, MA, USA; f Department of Pediatrics, Harvard Medical School, Boston, MA, USA; g School of Public Health, San Diego State University, San Diego, CA, USA; h Channing Division of Network Medicine, Harvard Medical School and Brigham and Women’s Hospital, Boston, MA, USA

**Keywords:** sexual minority, sexual orientation, health disparities, social discrimination, perceived discrimination

## Abstract

While discrimination has been studied among sexual minorities (SM), researchers have called for several necessary innovations, including adopting standard measures of discrimination and identifying attributions of such mistreatment among sexual minorities. We used data from the Nurses’ Health Study 2 (*n* = 69,483)—a longitudinal cohort of female nurses across the U.S. Experiences of discrimination were measured in 2017 using the Everyday Discrimination Scale (EDS) and coded using three schemes: situation, frequency, and chronicity. Participants attributed discriminatory experiences to any of 12 identities. We fit unadjusted quasi-Poisson models to estimate relative expected counts (RECs) for the association of sexual orientation with everyday discrimination and number of attributions, and unadjusted log-binomial models to estimate prevalence ratios (PRs) for specific attributions. Compared to participants who identified as completely heterosexual, all SM subgroups reported a higher number of situations and frequency of discrimination. Using chronicity coding, heterosexual with past same-sex attractions/partners/SM identity (REC:1.39, 95% CI:1.28–1.50), mostly heterosexual (REC:1.48, 95%CI:1.30–1.68), and bisexual (REC:1.57, 95%CI:1.13–2.11), but not lesbian (REC:1.00, 95%CI:0.78–1.26) participants reported more events of discriminatory experiences. All SM subgroups reported a greater number of attributions. Future research should use the EDS as a potential mediator in analyses of sexual orientation-related health disparities.

## Introduction

Over recent years, there has been growing interest in understanding the health of sexual minority people (e.g., those who identify as mostly heterosexual, bisexual, lesbian, etc., and/or those with same-sex/same-gender attraction or sexual partnerships). Researchers have identified health inequities in physical and mental health outcomes experienced by sexual minorities relative to their heterosexual counterparts (National Academies of Sciences, Engineering, and Medicine, 2020). One of the most commonly applied frameworks to explain sexual orientation-related health disparities is the Minority Stress Model, which asserts that sexual minorities experience a unique set of stressors related to anticipating, experiencing, internalizing, and coping with heterosexist stigma and discrimination (Brooks, 1981; Meyer, 2003). The biological effects of this chronic stress accumulate over time and result in worse physical and mental health outcomes (Dürrbaum & Sattler, 2020; Flentje et al., 2021). Sexual minorities may adopt coping strategies to alleviate stress, such as smoking tobacco and drinking alcohol, which may further contribute to adverse health outcomes. Lastly, sexual minorities may experience discrimination in synergy with other aspects of their identity (e.g., gender, racial identity, or body size), intensifying minority stress and potentially diversifying its consequences (Gordon et al., 2023; Raffoul et al., 2023; Walubita et al., 2022).

While discrimination has been studied among sexual minorities, researchers have called for several necessary innovations, including (1) using multiple dimensions of sexual orientation (i.e., identity, behavior, attraction), (2) adopting more standard/common measures of discrimination that capture multiple kinds of discriminatory experiences, and (3) identifying attribution of such mistreatment (Katz-Wise & Hyde, 2012). First, incorporating measurement of multiple dimensions of sexual orientation could help elucidate *who* is most at risk of experiencing discrimination, and potentially, *why*. Second, standardized discrimination measures facilitate comparison across studies and enable meta-analyses that could improve precision in estimates for small sexual minority subgroups (Logie et al., 2022; Semlyen, 2017). Finally, measurement of attributions can help clarify whether individuals were victimized based on sexual orientation or other dimensions of identity.

Research using the National Epidemiologic Survey on Alcohol and Related Conditions-III, a cross-sectional sample of over 36,309 US adults, has addressed some of these gaps. Studies using these data have incorporated measures of sexual identity, behavior, and attraction, and have demonstrated that experiences of discrimination mediate mental health and substance use related outcomes health outcomes (Krueger et al., 2020; Layland et al., 2020). However, the measure, adapted from the Experiences of Discrimination scale (Krieger et al., 2005), only assessed sexual orientation specific discrimination among sexual minority participants, which limits intercategorical comparisons.

The Nurses’ Health Study 2 (NHS2) is well-equipped to expand upon this research. In 2017, this large longitudinal cohort adopted measures of sexual orientation identity, sexual attraction, sexual partnerships, past sexual minority status, and everyday discrimination. “Everyday discrimination” refers to routine and sometimes subtle experiences of unfair treatment or discrimination. It is most commonly measured using the Everyday Discrimination Scale (EDS) (Bourabain & Verhaeghe, 2021; Williams et al., 1997), which asks how often the person experiences situations ranging from being treated with less courtesy or respect to being threated or harassed, among others. People who report any discrimination are then asked which aspects of their identity to which they most attribute these experiences. Research has demonstrated a consistent link between high EDS scores and poor mental health outcomes (Earnshaw et al., 2013; Everett et al., 2016; Pascoe & Smart Richman, 2009), and some research has found an association with adverse physical health (Cook et al., 2021; Earnshaw et al., 2013; Lawrence et al., 2022; Pascoe & Smart Richman, 2009), as well as health behaviors that might mediate health outcomes (Pascoe & Smart Richman, 2009). The NHS2 is one of few cohorts with such thorough measurement of both sexual orientation and discrimination experiences.

Using NHS2 data, we describe everyday discrimination using three coding schemes (i.e., number of situations, frequency, chronicity) across sexual orientation groups, as well as identify the type and number of identities to which participants attribute their experiences. We also describe everyday discrimination across sexual orientation by racialized group, ethnicity, body mass index (BMI), and employment as these may moderate or intensify experiences of discrimination while diversifying attributions (Walubita et al., 2022). It is critical to assess experiences at these intersections because interlocking systems of power and oppression (such as heterosexism, racism, ageism, sizeism, etc.) are mutually constitutive and therefore create unique lived experiences at each intersection (Bowleg, 2008; Crenshaw, 1990).

## Materials and methods

### Cohort

The Nurses’ Health Study 2 (NHS2) is a longitudinal cohort of registered nurses who self-identified as female at enrollment (*n* = 116,429) (Bao et al., 2016). The overall response rate to the baseline recruitment was 24%. Participants report detailed health information via online or paper questionnaire approximately every two years. Sixty five percent of participants returned the 2017 questionnaire. Participants were aged 24–44 years at enrollment in 1989 and aged 52–72 years in 2017. It is likely that most NHS2 participants are women, however, because gender identity has never been assessed in NHS2, we use gender-neutral terms to refer to participants (Rioux et al., 2022). Avoiding this form of misclassification is particularly important in the context of this study because sexual minority individuals are disproportionately likely to also identify as gender minorities (Reisner et al., 2023).

### Sexual orientation

In 2017, NHS2 participants were asked about their sexual orientation identity, prior identity, sexual attraction, and sexual behavior. To assess identity, participants were asked, “Which of the following best describes your feelings? Completely heterosexual (attracted to the opposite sex); mostly heterosexual; bisexual (equally attracted to men and women); mostly homosexual; completely homosexual (gay, lesbian, attracted to the same sex); not sure.” Participants were also asked if they had ever identified as “mostly heterosexual, bisexual, or lesbian or gay?” To assess attraction, participants were asked if they had ever been sexually attracted to females and (separately) whether they had ever been sexually attracted to males. Finally, to assess behavior, participants were asked whether they ever had sexual contact with males and/or females. Additionally, in 1995 and 2009, participants were asked, “Whether or not you are currently sexually active, what is your sexual orientation or identity? Heterosexual; lesbian, gay, or homosexual; bisexual; none of these; and prefer not to answer.”

Using 2017 data when available, we classified participants into one of the following five mutually exclusive groups: (1) completely heterosexual (reference), (2) heterosexual with past same-sex attractions/partners/identity, (3) mostly heterosexual, (4) bisexual, (5) lesbian (mostly or completely homosexual). Group 2 comprised participants who identified as completely heterosexual yet reported same-sex attraction, same-sex contact, and/or prior sexual minority identity; we omit the “completely” qualifier when describing heterosexual identity in this group to disambiguate them from the reference group. Seventeen respondents who reported that they were unsure of their sexual orientation identity were excluded due to small sample size.

Because the gender or sex of a person’s partners may influence how their sexual orientation is perceived by others and is associated with discriminatory experiences (Dyar et al., 2014; Hequembourg & Brallier, 2009; Ross et al., 2010), we additionally classify participants as (1) completely heterosexual with only male sexual partners, (2) heterosexual with female and male sexual partners, (3) heterosexual with only female sexual partners, (4) sexual minority (i.e., mostly heterosexual, bisexual, mostly homosexual, or completely homosexual) with only male sexual partners, (5) sexual minority with female and male sexual partners, and (6) sexual minority with only female sexual partners. We excluded individuals who reported no lifetime sexual partners from partnership analyses.

### Experiences of discrimination

Experiences of discrimination were measured using a modified version of the Everyday Discrimination Scale (EDS) (Williams et al., 1997). Prior research has found that this measure has good psychometric properties (e.g., acceptable Comparative Fit Index, high reliability score), including among sexual minority populations (Austin & Craig, 2013; Bastos et al., 2010; Gordon & Meyer, 2007). All participants were asked how often (i.e., almost every day, at least once/week, few times/month, few times/year, less than once/year, never) in their day-to-day life the following things happened, “You are treated with less courtesy or respect than other people; You receive poorer service than other people at restaurants or stores; People act as if they think you are not smart; People act as if they are afraid of you; People act as if they think you are dishonest; You are threatened or harassed.” The EDS does not specify an explicit timeframe.

Because > 98% of 2017 participants responded to all EDS scale questions, we restricted analyses to complete cases. Following a coding scheme described in detail elsewhere (Michaels et al., 2019), we categorized experiences of discrimination in three ways.

Situation-based coding counts the number of situations in which a participant ever experienced discrimination, ranging from 0–6 (e.g., someone who reported they were threatened or harassed almost every day but did not report other situations would be assigned a score of 1). Frequency-based coding converts the Likert scale to a numerical score (never = 0, almost every day = 5) and sums across items, ranging from 0–30 (e.g., someone who reported they were threatened or harassed almost every day but did not report other situations would be assigned a score of 5). Chronicity-based coding converts the frequencies reported on the Likert scale into an approximate number of events per year (almost every day = 260, at least once/week = 104, few times/month = 36, few times/year = 3, less than once/year = 0.5, never = 0) and sums across the six items, ranging from 0–1560 (e.g., someone who reported they were threatened or harassed almost every day but did not report other situations would be assigned a score of 260).

We considered these three coding schemes to facilitate comparison across approaches. Situation-based and frequency-based coding are the most commonly used measures (Michaels et al., 2019). Situation-based coding is conceptually clear: it reflects the number of discriminatory experience types that a person has had. Frequency-based coding attempts to capture *how often* people experience discrimination. However, by summing across the Likert-scale responses, frequency-based coding explicitly assumes the interval between response options is constant (i.e., the difference between almost every day and at least once/week is the same as the difference between less than once/year and never). Chronicity-based coding attempts to accurately determine the frequency of occurrence implied by each level of the Likert scale, thereby addressing the core flaw in frequency-based coding.

### Discrimination attribution

Participants were asked what they thought the main reasons were for their discriminatory experiences and were able to select all that apply from, “Your ancestry or national origins; Your gender; Your race; Your age; Your religion; Your height; Your weight; Some other aspect of your physical appearance; Your sexual orientation; Your education or income level; A physical disability; Other.” Among individuals who reported any discrimination, we calculated the total number of attributions.

### Covariates

Because the objective of this analysis is descriptive, we did not adjust for any variables (Lesko et al., 2022). However, in secondary analyses, we stratified by racialized group, ethnicity, BMI, age, and employment status. We categorized participants’ racialized group as white, Asian, Black or African, American Indian/Alaska Native, Native Hawaiian or Pacific Island, Multiracial, or Another/Unknown using items from the 1989 and 2005 questionnaires. Participants’ ethnicity was categorized as Hispanic/Latine or Non-Hispanic/Latine (Miranda et al., 2023). Using BMI in 2017 was categorized as <25, 25–<30, 30–<35, 35–<40, and 40+ kg/m^2^. Age at questionnaire return was categorized as 50–59, 60–69, and 70+ years. Self-reported employment status in 2017 was categorized as not employed (e.g. retired, disabled), nursing (e.g., nursing in a variety of hospital, outpatient, and community settings), and any other employment.

### Statistical analysis

We present the percentage of participants reporting each discriminatory situation, as well as the mean and standard deviation (SD) for the three coding schemes. Among those who report any discrimination, we report the percentage of participants reporting each attribution and the mean number of attributions. Given overdispersed data, we fit unadjusted quasi-Poisson models to estimate relative expected counts (i.e., ratio of the number of events) and 95% confidence intervals for the association between sexual orientation and both everyday discrimination and number of discrimination attributions (Gardner et al., 1995; Hayat & Higgins, 2014). We fit unadjusted log-binomial models to estimate prevalence ratios and corresponding 95% confidence intervals for the association between sexual orientation and endorsement of specific discrimination attributions (Luo et al., 2014). In secondary analyses, we included interaction terms between sexual orientation and either racialized group, ethnicity, BMI, or age. Finally, we repeated our main analysis using the different coding schemes for sexual partnerships.

## Results

Demographic characteristics are shown in Table 1. Of the 69,483 participants in our analytic sample, 88.9% identified as completely heterosexual, 7.1% were heterosexual with past same-sex attractions/partners/identity, 2.6% were mostly heterosexual, 0.4% were bisexual, and 1.1% were lesbian. The majority of the sample was white (94.8%) and not Hispanic/Latine (98.4%). The most reported type of everyday discrimination was being treated with less courtesy or respect than other people, which was reported by more than half of the participants in all sexual orientation subgroups (Table 2). Completely heterosexual participants reported the fewest discriminatory situations (mean:1.7, standard deviation (SD):1.9), while mostly heterosexual (mean:2.5, SD:2.0) and bisexual (mean:2.3, SD:2.1) participants reported the most. Patterns were similar using frequency coding. Using chronicity coding, completely heterosexuals reported an average of 27 discriminatory events (mean:26.9, SD:88.9), heterosexuals with past same-sex attractions/partners/identity 37 events (mean:37.3, SD:104.7), mostly heterosexuals 40 events (mean:39.8, SD:106.9), bisexuals reported the most at 42 events (mean:42.2, SD:116.6), and lesbians the least at 27 events (mean:26.8, SD:81.4).

Among those who experienced everyday discrimination, the most common attribution was age for completely heterosexual, heterosexual with past same-sex attractions/partners/identity, and mostly heterosexual participants, while gender was the most common for bisexual and lesbian participants. Completely heterosexual participants reported the fewest number of attributions (mean:1.5, SD:0.9) and bisexual participants reported the greatest number of attributions (mean:1.9, SD:1.2), on average.

All sexual minority subgroups reported a higher number of situations and frequency of discrimination, compared to completely heterosexuals (Table 3). For example, mostly heterosexual participants reported 1.43-fold (95%CI:1.37–1.49) the number of discriminatory situations compared to completely heterosexuals. Using chronicity coding, heterosexuals with past same-sex attractions/partners/identity (relative expected count [REC]:1.39, 95%CI:1.28–1.50), mostly heterosexual (REC:1.48, 95%CI:1.30–1.68), and bisexual (REC:1.57, 95%CI:1.13–2.11) participants reported substantially more events of discrimination than completely heterosexuals. However, lesbian participants were no different from completely heterosexual participants using chronicity coding (REC:1.00, 95%CI:0.78–1.26).

Heterosexual with past same-sex attractions/partners/identity (REC: 1.05, 95%CI:1.02–1.07), mostly heterosexual (REC:1.16, 95% CI:1.12–1.19), bisexual (REC:1.26, 95%CI:1.16–1.36), and lesbian (REC:1.25, 95%CI:1.19–1.31) participants reported a greater number of attributions than completely heterosexual participants. Bisexual (prevalence ratio [PR]:11.28, 95%CI:6.89–17.17) and lesbian (PR:40.50, 95% CI:34.25–47.74) participants were substantially more likely to attribute their discriminatory experiences to their sexual orientation than completely heterosexual participants. Mostly heterosexual (PR:1.38, 95% CI:1.29–1.47), bisexual (PR:1.65, 95%CI:1.42–1.89), and lesbian (PR:1.58, 95%CI:1.44–1.73) participants, but not heterosexual with past same-sex attractions/partners/identities (PR:1.04, 95%CI:0.99–1.10), were also more likely than completely heterosexuals to attribute discrimination to their gender.

Across racialized group (Supplementary Table S1), ethnicity (Supplementary Table S2), and BMI (Supplementary Table S3), we observed that participants belonging to multiply marginalized groups reported a higher prevalence of situation, frequency, and chronicity of discrimination compared to multiply privileged reference groups. Notably, racially minoritized mostly heterosexual and bisexual participants reported markedly higher discrimination chronicity compared to white completely heterosexuals. Additionally, in most sexual orientation subgroups, a dose-response relationship between BMI and discrimination chronicity was apparent, with those in the highest BMI categories reporting the greatest chronicity. Across age (Supplementary Table S4), younger participants reported more discrimination than older participants. Participants working in nursing generally reported the highest levels discrimination across all three coding schemes (Supplementary Table S5). Notably, many of these subgroups were small, and we lacked power to detect statistically significant effects for many of the multiply marginalized groups.

When we stratified by sexual orientation and partnership, sexual minority participants with only male sexual partners reported the most discrimination relative to completely heterosexuals with only male partners using all coding schemes (Supplementary Table S6). Relative to completely heterosexuals with only male partners, sexual minority participants with both male and female sexual partners (PR:18.2, 95%CI:14.98–21.96) and sexual minority participants with only female sexual partners (PR:37.74, 95%CI:29.51–47.20) attributed the discrimination they experienced to their sexual orientation most often.

## Discussion

Using a large cohort of female nurses, we found that sexual minorities experienced everyday discrimination in more situations, and that this discrimination occurred more frequently, as compared to completely heterosexuals. We also found that bisexuals and lesbians were dramatically more likely to attribute the discrimination they experienced to their sexual orientation, and mostly heterosexual, bisexual, and lesbian participants were more likely to attribute the discrimination they experienced to their gender as compared to completely heterosexuals.

The overall prevalence of reported everyday discrimination was larger in our study than we anticipated based on the existing literature. For example, one systematic review found that 41% of lesbian, gay, and bisexual people reported they had experienced discrimination (Katz-Wise & Hyde, 2012), while nearly two-thirds of sexual minorities in our study reported experiencing at least one discriminatory situation in their day-to-day life. This discrepancy may be due to the age of the data included in the systematic review (1988–2006), differing measures of discrimination, varying time frames (e.g., current, past-year, lifetime), or the unique demographic composition of the NHS2 cohort (e.g., female nurses, older, predominantly white). However, studies consistently show that discrimination is more common among sexual minority people than heterosexuals, which we observed for frequency and situation coding (Everett et al., 2016; Katz-Wise & Hyde, 2012; Scheim & Bauer, 2019).

However, when we used chronicity coding, we found that lesbians did not differ from completely heterosexuals in the number of days they reported experiencing discriminatory events (REC:1.00, 95%CI:0.78–1.26). This discrepancy between situation/frequency and chronicity coding is striking and indicates that lesbian participants reported a wider range of discriminatory experiences while also reporting that the frequency of discrimination happens as often as completely heterosexuals.

We found that lesbian participants were 40-times as likely to attribute the discrimination they experienced to sexual orientation as completely heterosexuals, and that bisexuals were 11-times as likely to do so. While striking, other studies have found similar or larger associations. For example, one study found that sexual and gender minority people were over 200-times as likely to attribute the discrimination they experienced to sexual orientation as cisgender heterosexuals (Godley, 2018). Another study found that lesbians were substantially more likely to attribute the discrimination they experienced to sexual orientation than bisexuals, but that bisexuals were more likely to report other attributions, similar to what we observed (Kiekens et al., 2022). While smaller in magnitude, we also found that mostly heterosexual, bisexual, and lesbian participants were more likely than completely heterosexuals to attribute the discrimination they experienced to gender. This is in line with research that has found a substantial proportion of sexual minority individuals attribute the discrimination they experience to gender-nonconformity (Gordon & Meyer, 2007), and is consistent with the intersectional nature of sexism and heterosexism in the production of heteropatriarchy (Everett et al., 2021). Finally, we found that sexual minorities with additional marginalized identities reported more discrimination and attributed this discrimination to a greater number of identities compared to white completely heterosexuals. This corroborates prior research demonstrating an increased prevalence of discrimination experiences among multiply marginalized individuals (Harnois, 2014; Walubita et al., 2022).

Our study has some limitations. Our measures of attraction and partnership use binary sex-based terminology (i.e., male and female), rather than gender-based terminology, and are not inclusive of attraction to or sexual partnership with non-binary individuals. This is particularly important because sexual minorities are disproportionately likely to themselves be or to partner with transgender and non-binary people, and vice versa (Reisner et al., 2023) and because gender expression is often more visible than sexual identity. Additionally, we know from participants’ reported sex assigned at birth that our sample experiences sex- and gender-based oppression. Future research on discrimination among sexual minorities should disaggregate results across gender identity with particular attention to the experiences of sexual minority women. However, the lack of gender identity measurement in NHS2 prevents our paper from contributing in this way.

This sample overrepresents participants who benefit from racial (predominantly white) and class privilege (nurses), and, thus, their experiences may not reflect those of the broader population. For example, nurses often work in dynamic teams involving a wide variety of contacts with patients, supervisors, other colleagues that may make their experience and appraisal of discrimination distinct from those other workplaces (Eliason et al., 2011; Mohr et al., 2025). Because participants were, on average, in their early 60s at the time of EDS completion, the findings may be less transferable to younger cohorts. Additionally, while the EDS is one of the most used measures in research on everyday discrimination, this measure has some limitations. First, the EDS aims to measure daily, interpersonal discrimination (Bourabain & Verhaeghe, 2021; Essed, 1991), rather than structural forms of injustice that someone may not perceive or be aware of (such as hiring and housing-related discrimination) (Bastos et al., 2017). In future research, the EDS could be used in concert with structural measures of discrimination to more fully capture how multi-level discrimination patterns health (Krieger, 2020). Notably, the EDS does not capture participants’ reaction to discrimination nor skills or support to cope with discrimination, which would better contextualize why we found the discrimination patterning described here.

Further, the question text for the EDS does not name “discrimination” and may actually capture events where people feel they experienced general mistreatment, rather than targeted discrimination (Bastos et al., 2017; Harnois et al., 2019). An advantage of this phrasing is the prevention of systematic underreporting of discrimination due to attributional ambiguity whereby a person may be uncertain as to whether an event was discriminatory (Bastos et al., 2017; Major & Crocker, 1993; Major et al., 2002) However, social identity may moderate whether an event or experience is perceived as discrimination or mistreatment (Bourabain & Verhaeghe, 2021), with differential item functioning across social identity (Lewis et al., 2012), and in turn, perceived discrimination and mistreatment may have different health impacts (Bastos et al., 2017). For example, non-Latine white people are more likely than people marginalized across axes of racialized identities and ethnicities to interpret the scale as referring to negative interactions rather than social inequality or discrimination (Harnois, 2022). Additionally, the EDS does not explicitly specify a time-frame for reports of discrimination, limiting its interpretability and comparability to other measures.

Lastly, while the EDS was originally developed to assess differences in experiences of discrimination across race, the scale intentionally used a framing of unfairness rather than racism (Williams et al., 1997). However, the chosen items in the scale may be more salient to the experiences of anti-Black racism and discrimination (Gee et al., 2009; Scheim & Bauer, 2019) and may not fully capture common experiences of gender or sexual orientation-based discrimination (e.g., concealment of sexual orientation, family exclusion, exclusion from gender-segregated spaces/services.)

More recently, new measures have been developed to capture a wide array of discriminatory experiences across many axes of social marginalization, such as the Intersectional Discrimination Index, which will provide additional information for researchers conducting intercategorical intersectional research (McCall, 2005; Scheim & Bauer, 2019).

Despite these limitations, our study has several strengths. First, the large analytic sample size of 69,483 participants and multidimensional measures of sexual orientation identity, attraction, and behavior allowed us to disaggregate sexual minority subgroups such as heterosexuals with past same-sex attractions/partners/identity and mostly heterosexuals. These groups reported elevated levels of discrimination across all three coding schemes. Therefore, studies that aggregate these groups into the reference group may underestimate sexual orientation-related disparities in discrimination. Our analytic sample size also allowed us to explore intersectional experiences of discrimination along axes of race, ethnicity, weight, and age, highlighting important intragroup variability in experiences of discrimination.

Our analysis also uses multiple coding schemes for everyday discrimination, which allows us to make comparisons and establishes a precedent for future research using this scale. For example, we recommend that future research using the EDS employ situation-based and/or chronicity-based coding but not frequency coding. Both situation and chronicity coding schemes are conceptually clear, while frequency-based coding makes the implausible assumption that the EDS response options are equally spaced (i.e., a 1-point difference using frequency coding could be the difference between almost every day and at least once per week or the difference between less than once per year and never). Because most people in this study reported frequencies at the lower end of the scale, frequency is a rough proxy for situation. Indeed, situation and frequency coding produced very similar results in our analysis, and the two measures are highly correlated (correlation:0.87). Chronicity is conceptually distinct from both, in that this coding explicitly attempts to measure how chronic discriminatory experiences are by delineating plausible event frequencies. This is a crucial distinction because researchers often hypothesize that discrimination impacts health through chronic exposure and biological dysregulation (Michaels et al., 2019). Therefore, we recommend that future research assessing how EDS mediates the relationship between social positioning and adverse health outcomes use a chronicity-based coding, as it is most aligned with the mechanisms through which discrimination is assumed to impact health.

Additionally, while we used continuous coding of the EDS, future research on the relationship between EDS and health could employ a categorical coding (e.g., low, moderate, high) based on the distribution of scores in a sample to improve interpretability (Beccia et al., 2020; Michaels et al., 2019) or could allow for a non-linear relationship between quantitative coded measures and the health outcome of interest, to avoid making the assumption that discrimination has a linear effect on health. While we assessed experiences of discrimination at the intersection of multiple identities, future work could explicitly assess effect modification by multiple identities, that is considering the unique effect discrimination has on those in identity subgroups across racial, gender, and sexual orientation categories. This could prove fruitful as interlocking axes of social inequality may interact to produce unique intersectional disparities that differ from the effects of each identity in isolation (Bowleg, 2008, 2012; Crenshaw, 1990).

We are currently collecting new data among sexual minority participants in another cohort, the Nurses’ Health Study 3, including measures drawn from the Daily Heterosexist Experiences Questionnaire (Balsam et al., 2013) that focus on vigilance and identity concealment, vicarious trauma, parenting, as well as additional measures on outness, LGBTQ social engagement and community. These new measures will complement the existing research by extending our understanding of potential mediators of these effects.

Finally, the majority of research on everyday discrimination and health is cross-sectional (Bourabain & Verhaeghe, 2021). Future research could use the NHS2 cohort, and similar cohorts, to assess whether EDS mediates the development of sexual orientation-related disparities in adverse health over time. Because research has found that the relationship between perceived discrimination and health varies across social identity groups (Everett et al., 2016; Schmitt et al., 2014), research using the EDS as a mediator of sexual orientation-related health disparities should allow for exposure-mediator interaction (i.e., effect modification).

In conclusion, we found that sexual minority participants reported experiencing everyday discrimination in more situations, at higher frequency, and in more events (except for lesbians who reported an equivalent number of events) as compared to completely heterosexual participants. We also demonstrated an increased burden of discrimination among sexual minorities with multiply marginalized identities. Finally, sexual minorities reported more attributions underlying their experiences and were substantially more likely to attribute the discrimination they experienced to their sexual orientation and gender than completely heterosexual participants. Our finding that lesbians and heterosexual participants did not differ in the chronicity of discriminatory events is a novel one that should be explored in subsequent research. Given the unique appraisal of discrimination attribution among this group, further psychometric evaluation of the EDS measure is warranted to explain this result. Our finding of more discrimination among participants who were heterosexual with past same-sex attractions/partners/sexual minority identity, as well as among mostly heterosexual participants, demonstrates the importance of gathering nuanced sexual orientation data. Future studies should disaggregate sexual minorities who identify as heterosexual but who report same-sex attractions or partners from the reference group to avoid underestimating disparities. Future research should extend the application of the EDS scale to examine discrimination as a potential mediator in analyses of sexual orientation-related health disparities, particularly among multiple marginalized sexual minority subgroups.

## Supplementary Material

Supp 1

Supplemental data for this article can be accessed online at https://doi.org/10.1080/00918369.2026.2635574

## Figures and Tables

**Table 1.** Demographic characteristics of eligible participants in the nurses’ health study 2 across sexual orientation (*N* = 69,484).

	Completely Heterosexual (*n* = 61738, 88.9%)	Heterosexual with Past Same-Sex Attractions/Partners/Identity (*n* = 4908, 7.1%)	Mostly Heterosexual (*n* = 1807, 2.6%)	Bisexual (*n* = 282, 0.4%)	Lesbian (*n* = 748, 1.1%)

Age in 2017 (mean, SD)	62.7 (4.6)	62.9 (4.5)	62.4 (4.5)	63.3 (4.8)	63.3 (4.4)
Racialized group					
American Indian or Alaska Native	35 (0.1%)	3 (0.1%)	2 (0.1%)	0 (0.0%)	0 (0.0%)
Asian or Pacific Islander	706 (1.1%)	150 (3.1%)	24 (1.3%)	3 (1.1%)	8 (1.1%)
Black	655 (1.1%)	107 (2.2%)	18 (1.0%)	1 (0.4%)	8 (1.1%)
Multiracial	1,068 (1.7%)	110 (2.2%)	49 (2.7%)	11 (3.9%)	14 (1.9%)
White	58,758 (95.2%)	4,471 (91.1%)	1,701 (94.1%)	263 (93.3%)	713 (95.3%)
Another or unknown	516 (0.8%)	67 (1.4%)	13 (0.7%)	4 (1.4%)	5 (0.7%)
Ethnicity					
Not Hispanic/Latine	60,800 (98.5%)	4,780 (97.4%)	1,759 (97.3%)	272 (96.5%)	740 (98.9%)
Hispanic/Latine	938 (1.5%)	128 (2.6%)	48 (2.7%)	10 (3.5%)	8 (1.1%)
Body Mass Index					
<25	23,716 (38.4%)	1,749 (35.6%)	685 (37.9%)	84 (29.8%)	226 (30.2%)
25–<30	18,470 (29.9%)	1,525 (31.1%)	539 (29.8%)	81 (28.7%)	211 (28.2%)
30–<35	10,066 (16.3%)	815 (16.6%)	294 (16.3%)	53 (18.8%)	153 (20.5%)
35–<40	4,439 (7.2%)	379 (7.7%)	130 (7.2%)	33 (11.7%)	78 (10.4%)
≥40	2,700 (4.4%)	239 (4.9%)	88 (4.9%)	24 (8.5%)	47 (6.3%)
Missing	2,347 (3.8%)	201 (4.1%)	71 (3.9%)	7 (2.5%)	33 (4.4%)
Employment					
Not Working	28,092 (45.5%)	2,266 (46.2%)	726 (40.2%)	140 (49.6%)	408 (54.5%)
Nursing	25,401 (41.1%)	2,018 (41.1%)	810 (44.8%)	104 (36.9%)	248 (33.2%)
Other employment	4,884 (7.9%)	337 (6.9%)	168 (9.3%)	26 (9.2%)	57 (7.6%)
Missing	3,361 (5.4%)	287 (5.8%)	103 (5.7%)	12 (4.3%)	35 (4.7%)

**Table 2.** Occurrence and attribution of discrimination among eligible participants in the nurses’ health study 2 across sexual orientation (*N* = 69,484).

	Completely Eleterosexual	Eleterosexual with Past Same-Sex Attractions/Partners/ldentity	Mostly Eleterosexual	Bisexual	Lesbian

	(*n* = 61738, 88.9%)	(*n* = 4908, 7.1%)	(*n* = 1807, 2.6%)	(*n* = 282, 0.4%)	(*n* = 748, 1.1%)

Discrimination Type (Ever, n, %)					
You are treated with less courtesy or respect than other people	32,083 (52.0%)	2,896 (59.0%)	1,173 (64.9%)	174 (61.7%)	446 (59.6%)
You receive poorer service than other people at restaurants or stores	23,499 (38.1%)	2,172 (44.3%)	898 (49.7%)	130 (46.1%)	359 (48.0%)
People act as if they think you are not smart	22,261 (36.1%)	2,050 (41.8%)	920 (50.9%)	123 (43.6%)	295 (39.4%)
People act as if they are afraid of you	10,178 (16.5%)	1,112 (22.7%)	505 (27.9%)	67 (23.8%)	166 (22.2%)
People act as if they think you are dishonest	6,712 (10.9%)	728 (14.8%)	365 (20.2%)	53 (18.8%)	112 (15.0%)
You are threatened or harassed Coded Measures (mean, SD)	12,045 (19.5%)	1,241 (25.3%)	602 (33.3%)	91 (32.3%)	220 (29.4%)
Situation	1.73 (1.85)	2.08 (1.96)	2.47 (2.00)	2.26 (2.07)	2.14 (1.94)
Frequency	2.99 (3.75)	3.75 (4.20)	4.39 (4.28)	4.10 (4.50)	3.71 (3.99)
Chronicity	26.88 (88.85)	37.26 (104.72)	39.8 (106.92)	42.22 (116.61)	26.84 (81.37)

	(*n* = 34160, 87.1%)	(*n* = 3092, 7.9%)	(*n* = 1295, 3.3%)	(*n* = 186, 0.5%)	(*n* = 498, 1.3%)

Attribution (n, %)					
Age	13,777 (40.3%)	1,206 (39.0%)	609 (47.0%)	85 (45.7%)	195 (39.2%)
Ancestry or national origins	1,082 (3.2%)	170 (5.5%)	57 (4.4%)	6 (3.2%)	17 (3.4%)
Education or income level	2,782 (8.1%)	261 (8.4%)	119 (9.2%)	11 (5.9%)	30 (6.0%)
Gender	10,545 (30.9%)	993 (32.1%)	550 (42.5%)	95 (51.1%)	243 (48.8%)
Height	1,441 (4.2%)	139 (4.5%)	72 (5.6%)	11 (5.9%)	16 (3.2%)
Physical appearance, some other aspect	1,736 (5.1%)	201 (6.5%)	94 (7.3%)	14 (7.5%)	30 (6.0%)
Physical disability	906 (2.7%)	128 (4.1%)	51 (3.9%)	13 (7.0%)	20 (4.0%)
Racialized group	1,920 (5.6%)	267 (8.6%)	79 (6.1%)	18 (9.7%)	32 (6.4%)
Religion	792 (2.3%)	64 (2.1%)	23 (1.8%)	5 (2.7%)	4 (0.8%)
Sexual orientation	293 (0.9%)	37 (1.2%)	17 (1.3%)	18 (9.7%)	173 (34.7%)
Weight	4,075 (11.9%)	377 (12.2%)	206 (15.9%)	32 (17.2%)	79 (15.9%)
Other	13,121 (38.4%)	1,121 (36.3%)	427 (33.0%)	52 (28.0%)	114 (22.9%)
Number of Attributions (mean, SD)	1.54 (0.92)	1.61 (0.97)	1.78 (1.09)	1.94 (1.17)	1.91 (1.14)

**Table 3.** Occurrence and attribution of discrimination among eligible participants in the nurses’ health study 2 compared across sexual orientation (*N* = 69,484).

	Completely Heterosexual	Heterosexual with Past Same-Sex Attractions/Partners/Identity	Mostly Heterosexual	Bisexual	Lesbian

Situation^1^	Ref.	1.20 (1.17–1.24)	1.43 (1.37–1.49)	1.31 (1.17–1.46)	1.24 (1.15–1.32)
Frequency^1^	Ref.	1.25 (1.21–1.30)	1.47 (1.40–1.54)	1.37 (1.21–1.55)	1.24 (1.14–1.34)
Chronicity^1^	Ref.	1.39 (1.28–1.50)	1.48 (1.30–1.68)	1.57 (1.13–2.11)	1.00 (0.78–1.26)
Among those who reported discrimination					
Attributions^1^	Ref.	1.05 (1.02–1.07)	1.16 (1.12–1.19)	1.26 (1.16–1.36)	1.25 (1.19–1.31)
Sexual orientation^2^	Ref.	1.40 (0.98–1.93)	1.53 (0.91–2.41)	11.28 (6.89–17.17)	40.50 (34.25–47.74)
Gender^2^	Ref.	1.04 (0.99–1.10)	1.38 (1.29–1.47)	1.65 (1.42–1.89)	1.58 (1.44–1.73)

a Relative expected counts and

2 prevalence ratios.

## Data Availability

Further information including the procedures to obtain and access data from the Nurses’ Health Study 2 is described at https://www.nurseshealthstudy.org/researchers (contact e-mail: nhsaccess@channing.harvard.edu).
