# Supplementary material for: Experiences of Everyday Discrimination by Sexual Orientation in a Large National Cohort of Female Nurses
Source: J Homosex. Author manuscript; Available in PMC 2026 Apr 7. (PMC13054939; doi:10.1080/00918369.2026.2635574)
Supplement: Supp 1 [file NIHMS2153871-supplement-Supp_1.docx]

# Supplementary Tables

| Supplemental Table 1. Occurrence and attribution of discrimination among eligible participants in the Nurses’ Health Study 2 compared across sexual orientation and racial group (N=69,484). | | | | | |  |
| --- | --- | --- | --- | --- | --- | --- |
|  | Completely Heterosexual | Heterosexual with Past Same-Sex Attractions/  Partners/Identity | Mostly Heterosexual | Bisexual | Lesbian | |
| White | | | | | |  |
| Situation^1^ | Ref. | 1.19 (1.15–1.22) | 1.42 (1.36–1.49) | 1.30 (1.15–1.45) | 1.25 (1.16–1.34) | |
| Frequency^1^ | Ref. | 1.24 (1.20–1.28) | 1.45 (1.38–1.53) | 1.33 (1.16–1.51) | 1.25 (1.15–1.35) | |
| Chronicity^1^ | Ref. | 1.37 (1.25–1.49) | 1.41 (1.23–1.62) | 1.40 (0.98–1.93) | 1.00 (0.77–1.26) | |
| Among those who reported discrimination | | | | | |  |
| Attributions^1^ | Ref. | 1.02 (1.00–1.05) | 1.15 (1.11–1.18) | 1.24 (1.14–1.34) | 1.25 (1.19–1.31) | |
| Sexual orientation^2^ | Ref. | 1.40 (0.95–1.98) | 1.40 (0.78–2.30) | 11.90 (7.16–18.32) | 42.70 (35.94–50.59) | |
| Race^2^ | Ref. | 1.16 (0.95–1.39) | 1.07 (0.79–1.41) | 2.16 (1.21–3.49) | 1.16 (0.71–1.74) | |
| Gender^2^ | Ref. | 1.04 (0.98–1.10) | 1.37 (1.28–1.47) | 1.67 (1.43–1.91) | 1.60 (1.45–1.75) | |
| Racially Minoritized^‡^ | | | | | |  |
| Situation^1^ | 1.37 (1.32–1.42) | 1.59 (1.48–1.72) | 1.85 (1.62–2.12) | 1.53 (0.94–2.47) | 1.33 (0.97–1.82) | |
| Frequency^1^ | 1.43 (1.37–1.49) | 1.67 (1.51–1.84) | 2.17 (1.79–2.61) | 2.34 (1.27–4.31) | 1.52 (1.01–2.29) | |
| Chronicity^1^ | 1.66 (1.49–1.85) | 1.92 (1.45–2.54) | 3.53 (2.27–5.48) | 5.17 (2.25–11.87) | 1.53 (0.70–3.34) | |
| Among those who reported discrimination | | | | | |  |
| Attributions^1^ | 1.30 (1.26–1.33) | 1.37 (1.28–1.47) | 1.57 (1.35–1.82) | 1.65 (0.99–2.76) | 1.52 (1.13–2.05) | |
| Sexual orientation^2^ | 1.56 (0.98–2.34) | 2.06 (0.85–4.96) | 4.52 (1.46–13.99) | 12.04 (1.52–95.39) | 26.18 (11.54–59.41) | |
| Race^2^ | 12.34 (11.39–13.35) | 13.88 (12.17–15.83) | 11.10 (8.35–14.75) | 8.59 (3.00–24.64) | 14.94 (9.88–22.58) | |
| Gender^2^ | 1.15 (1.08–1.23) | 1.15 (0.98–1.34) | 1.51 (1.19–1.92) | 1.31 (0.56–3.03) | 1.56 (1.00–2.44) | |
|  | white | Asian or Pacific Islander | American Indian or Alaska Native | Black | Multiracial | |
| Completely Heterosexual | | | | | |  |
| Situation^1^ | Ref. | 1.35 (1.26–1.45) | 1.41 (1.03–1.88) | 1.65 (1.54–1.75) | 1.21 (1.14–1.28) | |
| Frequency^1^ | Ref. | 1.27 (1.17–1.38) | 1.37 (0.94–1.92) | 1.82 (1.69–1.95) | 1.28 (1.20–1.37) | |
| Chronicity^1^ | Ref. | 1.07 (0.83–1.35) | 1.77 (0.68–3.67) | 2.44 (2.06–2.87) | 1.58 (1.34–1.84) | |
| Among those who reported discrimination | | | | | |  |
| Attributions^1^ | Ref. | 1.35 (1.29–1.42) | 1.19 (0.94–1.47) | 1.40 (1.34–1.47) | 1.18 (1.13–1.23) | |
| Sexual orientation^2^ | Ref. | 1.51 (0.60–3.08) | – | 2.30 (1.15–4.06) | 1.07 (0.43–2.19) | |
| Race^2^ | Ref. | 13.06 (11.62–14.59) | – | 22.41 (20.81–24.09) | 4.17 (3.42–5.02) | |
| Gender^2^ | Ref. | 1.19 (1.05–1.33) | – | 1.09 (0.96–1.23) | 1.18 (1.06–1.30) | |
| Sexual Minority^†^ | | | | | |  |
| Situation^1^ | 1.25 (1.23–1.28) | 1.66 (1.51–1.84) | – | 1.73 (1.55–1.94) | 1.50 (1.34–1.69) | |
| Frequency^1^ | 1.30 (1.26–1.33) | 1.71 (1.49–1.96) | – | 1.94 (1.65–2.27) | 1.70 (1.47–1.98) | |
| Chronicity^1^ | 1.34 (1.25–1.44) | 2.09 (1.36–3.23) | – | 2.58 (1.69–3.94) | 2.24 (1.64–3.06) | |
| Among those who reported discrimination | | | | | |  |
| Attributions^1^ | 1.09 (1.07–1.11) | 1.48 (1.34–1.64) | – | 1.51 (1.36–1.69) | 1.31 (1.16–1.47) | |
| Sexual orientation^2^ | 6.02 (5.06–7.16) | 4.07 (1.70–9.77) | – | 1.08 (0.15–7.73) | 6.83 (3.43–13.60) | |
| Race^2^ | 1.17 (1.00–1.36) | 13.54 (11.31–16.22) | – | 23.52 (21.19–26.11) | 5.28 (3.71–7.53) | |
| Gender^2^ | 1.21 (1.16–1.26) | 1.17 (0.94–1.45) | – | 1.31 (1.04–1.65) | 1.32 (1.08–1.62) | |
| ^1^Relative expected counts and ^2^prevalence ratios.  **^†^**Heterosexual with past same-sex attractions/partners/identity, Mostly Heterosexual, Bisexual, or Lesbian  ^‡^Asian or Pacific Islander, American Indian or Alaska Native, Black, or Multiracial  Estimates excluded due small cell sizes (n<10) denoted with singular endash (–) | | | | | |  |

| Supplemental Table 2. Occurrence and attribution of discrimination among eligible participants in the Nurses’ Health Study 2 compared across sexual orientation and ethnicity (N=69,484). | | | | | |
| --- | --- | --- | --- | --- | --- |
|  | Completely Heterosexual | Heterosexual with Past Same-Sex Attractions/  Partners/Identity | Mostly Heterosexual | Bisexual | Lesbian |
| Not Hispanic/Latine | | | | | |
| Situation^1^ | Ref. | 1.20 (1.17–1.24) | 1.43 (1.37–1.49) | 1.30 (1.16–1.46) | 1.24 (1.15–1.32) |
| Frequency^1^ | Ref. | 1.25 (1.21–1.29) | 1.48 (1.40–1.55) | 1.38 (1.21–1.56) | 1.24 (1.14–1.35) |
| Chronicity^1^ | Ref. | 1.37 (1.26–1.49) | 1.51 (1.32–1.71) | 1.60 (1.15–2.16) | 1.00 (0.78–1.27) |
| Among those who reported discrimination | | | | | |
| Attributions^1^ | Ref. | 1.04 (1.02–1.07) | 1.16 (1.12–1.19) | 1.24 (1.15–1.35) | 1.25 (1.19–1.31) |
| Sexual orientation^2^ | Ref. | 1.39 (0.97–1.94) | 1.57 (0.93–2.47) | 10.97 (6.60–16.89) | 40.47 (34.19–47.75) |
| Ancestry or national origins^2^ | Ref. | 1.70 (1.43–2.01) | 1.35 (1.01–1.77) | 0.77 (0.24–1.77) | 1.13 (0.67–1.78) |
| Race^2^ | Ref. | 1.50 (1.31–1.70) | 1.06 (0.84–1.32) | 1.55 (0.90–2.41) | 1.13 (0.78–1.57) |
| Gender^2^ | Ref. | 1.04 (0.99–1.10) | 1.37 (1.28–1.46) | 1.62 (1.39–1.86) | 1.59 (1.44–1.73) |
| Hispanic/Latine | | | | | |
| Situation^1^ | 1.18 (1.10–1.25) | 1.34 (1.15–1.56) | 1.46 (1.15–1.85) | 1.51 (0.81–2.80) | – |
| Frequency^1^ | 1.23 (1.14–1.32) | 1.50 (1.24–1.81) | 1.45 (1.12–1.87) | 1.38 (0.67–2.81) | – |
| Chronicity^1^ | 1.44 (1.20–1.71) | 2.23 (1.54–3.24) | 0.90 (0.50–1.62) | 1.02 (0.21–5.02) | – |
| Among those who reported discrimination | | | | | |
| Attributions^1^ | 1.16 (1.11–1.22) | 1.20 (1.05–1.38) | 1.36 (1.13–1.64) | – | – |
| Sexual orientation^2^ | 0.80 (0.25–1.88) | 1.29 (0.18–9.30) | – | – | – |
| Ancestry or national origins^2^ | 7.04 (5.89–8.33) | 8.89 (6.19–12.77) | 7.95 (4.24–14.93) | – | – |
| Race^2^ | 3.53 (2.95–4.18) | 5.15 (3.67–7.24) | 3.71 (1.87–7.35) | – | – |
| Gender^2^ | 1.08 (0.96–1.21) | 1.01 (0.74–1.38) | 1.76 (1.29–2.41) | – | – |

^1^Relative expected counts and ^2^prevalence ratios.

Estimates excluded due small cell sizes (n<10) denoted with singular endash (–)

| Supplemental Table 3. Occurrence and attribution of discrimination among eligible participants in the Nurses’ Health Study 2 compared across sexual orientation and body mass index (BMI) (N=69,484). | | | | | |  |
| --- | --- | --- | --- | --- | --- | --- |
|  | Completely Heterosexual | Heterosexual with Past Same-Sex Attractions/  Partners/Identity | Mostly Heterosexual | Bisexual | Lesbian | |
| BMI <25 | | | | | |  |
| Situation^1^ | Ref. | 1.20 (1.15–1.27) | 1.49 (1.39–1.60) | 1.31 (1.06–1.60) | 1.22 (1.07–1.39) | |
| Frequency^1^ | Ref. | 1.25 (1.18–1.32) | 1.52 (1.40–1.65) | 1.28 (0.99–1.63) | 1.23 (1.05–1.44) | |
| Chronicity^1^ | Ref. | 1.33 (1.13–1.54) | 1.50 (1.19–1.87) | 0.92 (0.36–1.87) | 1.04 (0.63–1.60) | |
| Among those who reported discrimination | | | | | |  |
| Attributions^1^ | Ref. | 1.03 (0.99–1.07) | 1.16 (1.10–1.23) | 1.18 (1.00–1.37) | 1.21 (1.10–1.32) | |
| Sexual orientation^2^ | Ref. | 1.42 (0.78–2.37) | 1.60 (0.68–3.16) | 10.70 (3.91–22.29) | 31.51 (22.84–42.56) | |
| Weight^2^ | Ref. | 0.63 (0.31–1.13) | 2.72 (1.65–4.19) | 1.34 (0.08–5.74) | 1.37 (0.34–3.55) | |
| Gender^2^ | Ref. | 1.07 (0.98–1.17) | 1.41 (1.26–1.55) | 1.68 (1.25–2.10) | 1.60 (1.34–1.85) | |
| BMI 25–<30 | | | | | |  |
| Situation^1^ | 1.07 (1.05–1.10) | 1.30 (1.23–1.36) | 1.52 (1.42–1.63) | 1.55 (1.28–1.88) | 1.34 (1.18–1.52) | |
| Frequency^1^ | 1.11 (1.08–1.14) | 1.41 (1.33–1.50) | 1.61 (1.49–1.75) | 1.69 (1.33–2.14) | 1.45 (1.26–1.67) | |
| Chronicity^1^ | 1.22 (1.14–1.30) | 1.75 (1.51–2.02) | 1.75 (1.43–2.15) | 2.10 (1.12–3.96) | 1.35 (0.98–1.85) | |
| Among those who reported discrimination | | | | | |  |
| Attributions^1^ | 1.02 (1.00–1.04) | 1.06 (1.02–1.10) | 1.22 (1.14–1.30) | 1.33 (1.15–1.54) | 1.27 (1.16–1.39) | |
| Sexual orientation^2^ | 0.95 (0.72–1.25) | 1.80 (1.07–3.03) | 1.90 (0.89–4.06) | 11.67 (5.30–25.73) | 41.61 (31.61–54.78) | |
| Weight^2^ | 3.24 (2.75–3.84) | 3.59 (2.65–4.86) | 4.75 (3.23–6.99) | 4.86 (1.84–12.85) | 4.18 (2.18–8.03) | |
| Gender^2^ | 0.99 (0.95–1.03) | 1.00 (0.91–1.10) | 1.32 (1.18–1.49) | 1.69 (1.32–2.17) | 1.57 (1.34–1.86) | |
| BMI 30–<35 | | | | | |  |
| Situation^1^ | 1.16 (1.13–1.19) | 1.32 (1.23–1.40) | 1.64 (1.50–1.79) | 1.05 (0.77–1.42) | 1.33 (1.15–1.55) | |
| Frequency^1^ | 1.22 (1.18–1.25) | 1.44 (1.33–1.55) | 1.80 (1.62–2.01) | 1.18 (0.80–1.76) | 1.37 (1.15–1.63) | |
| Chronicity^1^ | 1.43 (1.32–1.54) | 1.77 (1.47–2.12) | 2.07 (1.53–2.79) | 2.42 (1.17–5.00) | 1.21 (0.79–1.86) | |
| Among those who reported discrimination | | | | | |  |
| Attributions^1^ | 1.11 (1.09–1.14) | 1.12 (1.07–1.18) | 1.22 (1.14–1.31) | 1.41 (1.08–1.84) | 1.36 (1.21–1.53) | |
| Sexual orientation^2^ | 1.05 (0.76–1.43) | 0.61 (0.19–1.91) | 0.49 (0.07–3.53) | 3.24 (0.44–23.93) | 37.90 (27.30–52.61) | |
| Weight^2^ | 13.28 (11.44–15.52) | 13.81 (11.07–17.21) | 17.79 (13.67–23.17) | 12.16 (5.69–26.00) | 13.24 (8.60–20.38) | |
| Gender^2^ | 0.94 (0.90–0.99) | 0.97 (0.85–1.10) | 1.33 (1.13–1.55) | 1.41 (0.96–2.08) | 1.55 (1.27–1.90) | |
| BMI 35–<40 |  |  |  |  |  | |
| Situation^1^ | 1.23 (1.19–1.27) | 1.51 (1.39–1.65) | 1.69 (1.49–1.91) | 1.70 (1.32–2.20) | 1.54 (1.27–1.85) | |
| Frequency^1^ | 1.31 (1.26–1.36) | 1.67 (1.51–1.84) | 1.87 (1.58–2.21) | 1.82 (1.39–2.38) | 1.65 (1.31–2.09) | |
| Chronicity^1^ | 1.51 (1.37–1.67) | 2.15 (1.69–2.75) | 3.00 (1.86–4.83) | 1.67 (0.82–3.41) | 1.62 (0.78–3.34) | |
| Among those who reported discrimination | | | | | |  |
| Attributions^1^ | 1.18 (1.15–1.21) | 1.30 (1.21–1.40) | 1.43 (1.27–1.59) | 1.57 (1.26–1.96) | 1.52 (1.28–1.80) | |
| Sexual orientation^2^ | 0.43 (0.22–0.76) | 0.41 (0.06–2.96) | 1.07 (0.15–7.74) | 22.29 (9.68–51.32) | 42.80 (29.37–62.37) | |
| Weight^2^ | 25.44 (21.93–29.68) | 24.95 (20.15–30.89) | 33.44 (26.19–42.70) | 36.22 (24.04–54.58) | 30.40 (21.90–42.19) | |
| Gender^2^ | 0.90 (0.85–0.96) | 0.98 (0.82–1.17) | 1.18 (0.92–1.52) | 1.81 (1.27–2.58) | 1.41 (1.05–1.90) | |
| BMI 40+ |  |  |  |  |  | |
| Situation^1^ | 1.35 (1.30–1.40) | 1.57 (1.42–1.73) | 1.77 (1.51–2.06) | 1.48 (1.04–2.11) | 1.67 (1.35–2.08) | |
| Frequency^1^ | 1.53 (1.46–1.59) | 1.85 (1.63–2.09) | 2.00 (1.66–2.42) | 1.88 (1.21–2.92) | 1.57 (1.23–2.01) | |
| Chronicity^1^ | 2.06 (1.85–2.29) | 2.74 (2.06–3.65) | 2.17 (1.26–3.71) | 3.57 (1.38–9.24) | 0.96 (0.49–1.88) | |
| Among those who reported discrimination | | | | | |  |
| Attributions^1^ | 1.22 (1.19–1.26) | 1.28 (1.17–1.40) | 1.32 (1.17–1.48) | 1.29 (0.94–1.77) | 1.50 (1.20–1.87) | |
| Sexual orientation^2^ | 0.18 (0.04–0.46) | 1.22 (0.30–4.92) | – | 6.29 (0.83–47.85) | 25.34 (13.72–46.82) | |
| Weight^2^ | 38.72 (33.48–45.08) | 35.72 (29.26–43.61) | 43.21 (34.25–54.51) | 31.47 (18.07–54.80) | 40.48 (29.98–54.65) | |
| Gender^2^ | 0.79 (0.72–0.85) | 0.65 (0.49–0.87) | 1.05 (0.74–1.49) | 1.10 (0.55–2.17) | 1.23 (0.82–1.84) | |
| ^1^Relative expected counts and ^2^prevalence ratios.  Estimates excluded due small cell sizes (n<10) denoted with singular endash (–) | | | | | |  |

| Supplemental Table 4. Occurrence and attribution of discrimination among eligible participants in the Nurses’ Health Study 2 compared across sexual orientation and age (N=69,484). | | | | | |
| --- | --- | --- | --- | --- | --- |
|  | Completely Heterosexual | Heterosexual with Past Same-Sex Attractions/  Partners/Identity | Mostly Heterosexual | Bisexual | Lesbian |
| 50–59 years | | | | | |
| Situation^1^ | Ref. | 1.18 (1.12–1.24) | 1.36 (1.27–1.47) | 1.33 (1.08–1.61) | 1.14 (0.98–1.31) |
| Frequency^1^ | Ref. | 1.20 (1.13–1.27) | 1.41 (1.30–1.53) | 1.39 (1.10–1.73) | 1.10 (0.93–1.30) |
| Chronicity^1^ | Ref. | 1.22 (1.04–1.42) | 1.47 (1.18–1.80) | 1.45 (0.77–2.45) | 0.89 (0.52–1.40) |
| Among those who reported discrimination | | | | | |
| Attributions^1^ | Ref. | 0.99 (0.94–1.03) | 1.17 (1.10–1.23) | 1.32 (1.13–1.53) | 1.16 (1.04–1.29) |
| Sexual orientation^2^ | Ref. | 1.56 (0.84–2.67) | 0.51 (0.08–1.61) | 10.37 (3.79–21.64) | 40.76 (29.81–54.69) |
| Age^2^ | Ref. | 0.86 (0.75–0.98) | 1.20 (1.03–1.38) | 1.48 (0.99–2.02) | 0.79 (0.52–1.10) |
| Gender^2^ | Ref. | 1.03 (0.92–1.13) | 1.41 (1.25–1.58) | 1.39 (0.96–1.84) | 1.39 (1.10–1.70) |
| 60–69 years | | | | | |
| Situation^1^ | 0.80 (0.78–0.81) | 0.96 (0.93–1.00) | 1.14 (1.09–1.20) | 1.04 (0.90–1.19) | 1.02 (0.95–1.11) |
| Frequency^1^ | 0.77 (0.75–0.78) | 0.98 (0.94–1.03) | 1.12 (1.06–1.19) | 1.04 (0.88–1.24) | 1.00 (0.91–1.10) |
| Chronicity^1^ | 0.71 (0.67–0.75) | 1.06 (0.95–1.18) | 0.99 (0.85–1.17) | 1.20 (0.77–1.87) | 0.77 (0.58–1.01) |
| Among those who reported discrimination | | | | | |
| Attributions^1^ | 1.02 (1.00–1.03) | 1.08 (1.05–1.11) | 1.18 (1.13–1.23) | 1.24 (1.11–1.39) | 1.29 (1.21–1.37) |
| Sexual orientation^2^ | 0.81 (0.63–1.03) | 1.00 (0.63–1.60) | 1.65 (0.95–2.88) | 8.29 (4.42–15.57) | 33.36 (26.14–42.57) |
| Age^2^ | 1.69 (1.63–1.75) | 1.64 (1.55–1.74) | 2.01 (1.87–2.15) | 1.78 (1.47–2.16) | 1.58 (1.39–1.79) |
| Gender^2^ | 0.98 (0.94–1.01) | 1.02 (0.95–1.09) | 1.35 (1.24–1.47) | 1.69 (1.43–2.01) | 1.58 (1.42–1.76) |
| 70+ years | | | | | |
| Situation^1^ | 0.61 (0.59–0.64) | 0.86 (0.76–0.97) | 1.05 (0.88–1.25) | 0.95 (0.66–1.37) | 0.89 (0.67–1.20) |
| Frequency^1^ | 0.55 (0.52–0.58) | 0.76 (0.66–0.88) | 1.04 (0.81–1.32) | 0.98 (0.68–1.43) | 0.85 (0.61–1.18) |
| Chronicity^1^ | 0.42 (0.36–0.48) | 0.53 (0.36–0.79) | 1.17 (0.64–2.14) | 0.65 (0.28–1.53) | 0.38 (0.20–0.71) |
| Among those who reported discrimination | | | | | |
| Attributions^1^ | 1.02 (0.99–1.05) | 1.12 (1.00–1.25) | 1.11 (0.97–1.28) | 1.39 (1.06–1.84) | 1.27 (1.07–1.52) |
| Sexual orientation^2^ | 0.64 (0.32–1.13) | 1.73 (0.55–5.42) | 1.56 (0.21–11.32) | 16.59 (5.46–50.39) | 27.46 (14.46–52.14) |
| Age^2^ | 2.16 (2.05–2.27) | 2.14 (1.88–2.45) | 1.93 (1.51–2.47) | 1.87 (1.15–3.06) | 2.58 (2.00–3.34) |
| Gender^2^ | 0.91 (0.84–0.98) | 1.01 (0.81–1.26) | 0.94 (0.64–1.38) | 1.76 (1.14–2.74) | 1.75 (1.25–2.46) |
| ^1^Relative expected counts and ^2^prevalence ratios. | | | | | |

| Supplemental Table 5. Occurrence and attribution of discrimination among eligible participants in the Nurses’ Health Study 2 compared across sexual orientation and employment status (N=69,484). | | | | | | |
| --- | --- | --- | --- | --- | --- | --- |
|  | Completely Heterosexual | Heterosexual with Past Same-Sex Attractions/  Partners/Identity | Mostly Heterosexual | Bisexual | Lesbian |  |
| Not Working | | | | | | |
| Situation^1^ | Ref. | 1.25 (1.19–1.31) | 1.59 (1.48–1.70) | 1.41 (1.20–1.65) | 1.34 (1.21–1.48) |  |
| Frequency^1^ | Ref. | 1.32 (1.25–1.39) | 1.66 (1.54–1.80) | 1.49 (1.23–1.79) | 1.40 (1.25–1.57) |  |
| Chronicity^1^ | Ref. | 1.55 (1.35–1.77) | 1.87 (1.50–2.29) | 1.89 (1.14–2.92) | 1.14 (0.79–1.60) |  |
| Among those who reported discrimination | | | | | | |
| Attributions^1^ | Ref. | 1.06 (1.02–1.09) | 1.15 (1.09–1.21) | 1.35 (1.21–1.50) | 1.26 (1.18–1.35) |  |
| Sexual orientation^2^ | Ref. | 1.71 (0.98–2.81) | 1.67 (0.66–3.47) | 15.94 (8.02–27.86) | 46.57 (35.80–60.47) |  |
| Gender^2^ | Ref. | 0.97 (0.89–1.05) | 1.32 (1.18–1.46) | 1.71 (1.37–2.04) | 1.62 (1.43–1.82) |  |
| Nursing | | | | | | |
| Situation^1^ | 1.34 (1.32–1.37) | 1.58 (1.52–1.64) | 1.76 (1.67–1.86) | 1.78 (1.51–2.09) | 1.55 (1.39–1.73) |  |
| Frequency^1^ | 1.44 (1.41–1.47) | 1.74 (1.66–1.82) | 1.93 (1.80–2.06) | 2.01 (1.66–2.42) | 1.69 (1.48–1.92) |  |
| Chronicity^1^ | 1.75 (1.66–1.86) | 2.25 (2.00–2.54) | 2.31 (1.92–2.78) | 2.78 (1.85–4.17) | 1.92 (1.33–2.76) |  |
| Among those who reported discrimination | | | | | | |
| Attributions^1^ | 0.97 (0.96–0.98) | 1.00 (0.97–1.04) | 1.09 (1.04–1.15) | 1.16 (1.02–1.32) | 1.18 (1.08–1.29) |  |
| Sexual orientation^2^ | 1.32 (1.02–1.70) | 1.64 (0.97–2.77) | 1.17 (0.48–2.88) | 14.96 (7.49–29.89) | 52.10 (39.56–68.60) |  |
| Gender^2^ | 0.97 (0.94–1.01) | 1.07 (0.99–1.16) | 1.32 (1.19–1.46) | 1.54 (1.21–1.95) | 1.44 (1.22–1.71) |  |
| Other Employment | | | | | | |
| Situation^1^ | 1.13 (1.10–1.17) | 1.35 (1.22–1.49) | 1.60 (1.40–1.82) | 1.20 (0.77–1.88) | 1.59 (1.28–1.97) |  |
| Frequency^1^ | 1.13 (1.08–1.17) | 1.42 (1.26–1.60) | 1.65 (1.41–1.92) | 1.20 (0.73–1.96) | 1.45 (1.13–1.85) |  |
| Chronicity^1^ | 1.10 (0.98–1.23) | 1.44 (1.09–1.91) | 1.26 (0.88–1.81) | 0.81 (0.26–2.52) | 0.84 (0.39–1.78) |  |
| Among those who reported discrimination | | | | | | |
| Attributions^1^ | 1.01 (0.99–1.04) | 1.02 (0.94–1.11) | 1.37 (1.20–1.56) | 1.18 (0.84–1.64) | 1.20 (1.00–1.45) |  |
| Sexual orientation^2^ | 1.25 (0.78–1.91) | 0.63 (0.09–4.57) | 6.15 (2.54–14.93) | – | 44.47 (26.97–73.34) |  |
| Gender^2^ | 1.10 (1.03–1.16) | 1.22 (1.03–1.44) | 1.66 (1.38–1.98) | 2.13 (1.38–3.30) | 1.87 (1.44–2.44) |  |
| ^1^Relative expected counts and ^2^prevalence ratios.  Estimates excluded due small cell sizes (n<10) denoted with singular endash (–) | | | | | | |

| Supplemental Table 6. Occurrence and attribution of discrimination among eligible participants in the Nurses’ Health Study 2 compared across sexual orientation and sexual partners (N=69,484). | | | | | | |  |
| --- | --- | --- | --- | --- | --- | --- | --- |
|  | Completely Heterosexual With Only Male Sexual Partners | Heterosexual With Both Male And Female Sexual Partners | Heterosexual With Only Female Sexual Partners | Sexual Minority With Only Male Sexual Partners | Sexual Minority With Both Male And Female Sexual Partners | Sexual Minority With Only Female Sexual Partners | |
| Situation^1^ | Ref. | 1.16 (1.06–1.27) | 1.21 (0.99–1.45) | 1.43 (1.37–1.50) | 1.29 (1.22–1.36) | 1.26 (1.12–1.41) | |
| Frequency^1^ | Ref. | 1.15 (1.03–1.28) | 1.34 (1.08–1.65) | 1.49 (1.41–1.57) | 1.30 (1.22–1.38) | 1.25 (1.09–1.42) | |
| Chronicity^1^ | Ref. | 1.06 (0.77–1.41) | 1.63 (0.94–2.61) | 1.53 (1.32–1.76) | 1.18 (0.99–1.40) | 1.06 (0.70–1.52) | |
| Among those who reported discrimination | | | | | | | |
| Attributions^1^ | Ref. | 1.08 (1.01–1.16) | 1.00 (0.86–1.17) | 1.19 (1.15–1.23) | 1.20 (1.15–1.24) | 1.19 (1.09–1.29) | |
| Sexual orientation^2^ | Ref. | 0.76 (0.13–2.35) | – | 1.82 (1.06–2.90) | 18.20 (14.98–21.96) | 37.74 (29.51–47.20) | |
| Gender^2^ | Ref. | 1.29 (1.11–1.47) | 0.77 (0.46–1.15) | 1.46 (1.36–1.56) | 1.47 (1.36–1.59) | 1.43 (1.20–1.67) | |
| ^1^Relative expected counts and ^2^prevalence ratios.  Estimates excluded due small cell sizes (n<10) denoted with singular endash (–) | | | | | | | |
